# Global disruption in excitation-inhibition balance can cause localized network dysfunction and Schizophrenia-like context-integration deficits

**DOI:** 10.1371/journal.pcbi.1008985

**Published:** 2021-05-25

**Authors:** Olivia L. Calvin, A. David Redish

**Affiliations:** 1 Department of Neuroscience, University of Minnesota, Minneapolis, Minnesota, United State of America; 2 Department of Psychiatry & Behavioral Sciences, University of Minnesota, Minneapolis, Minnesota, United State of America; McGill University, CANADA

## Abstract

Poor context integration, the process of incorporating both previous and current information in decision making, is a cognitive symptom of schizophrenia. The maintenance of the contextual information has been shown to be sensitive to changes in excitation-inhibition (EI) balance. Many regions of the brain are sensitive to EI imbalances, however, so it is unknown how systemic manipulations affect the specific regions that are important to context integration. We constructed a multi-structure, biophysically-realistic agent that could perform context-integration as is assessed by the dot pattern expectancy task. The agent included a perceptual network, a memory network, and a decision making system and was capable of successfully performing the dot pattern expectancy task. Systemic manipulation of the agent’s EI balance produced localized dysfunction of the memory structure, which resulted in schizophrenia-like deficits at context integration. When the agent’s pyramidal cells were less excitatory, the agent fixated upon the cue and initiated responding later than the default agent, which were like the deficits one would predict that individuals on the autistic spectrum would make. This modelling suggests that it may be possible to parse between different types of context integration deficits by adding distractors to context integration tasks and by closely examining a participant’s reaction times.

## Author summary

Schizophrenia is a debilitating mental health disorder and its underlying etiology is currently unknown. Neural imbalances in the neural excitation and inhibition of specific regions of the brain have been hypothesized to cause symptoms of schizophrenia. Most regions of the brain have specific excitation-inhibition balances that permit their functioning in the processing of information. How systemic changes in the excitation-inhibition balance cause specific deficits and dysfunction within neural circuits is unknown. A common cognitive deficit in schizophrenia is difficulty with context integration, which is the ability to successfully use previous and current information when making decisions. We assessed how this symptom could be caused by an imbalance in neural excitation and inhibition by simulating the effects of potential imbalances in a model agent. Global imbalances in the agent’s neural excitation and inhibition led to impairment of specific circuits. These dysfunctional circuits produced behavioral deficits that were like those observed in individuals with schizophrenia. These simulations suggested how specific neural circuits may be disrupted by global changes in excitation or inhibition, ways to improve the assessment of context integration, new approaches to analyzing behavior, and why it may be beneficial to assess context integration in autism spectrum disorder.

## Introduction

Schizophrenia is a debilitating psychiatric disorder that can be devastating to the individuals who suffer from it, to their families, and to society. While psychiatric treatments have been developed that alleviate symptoms of schizophrenia, its etiology is currently unknown [1]. A recent hypothesis is that an excitation-inhibition (EI) neural imbalance causes the symptoms of schizophrenia [2–4]. Genetic and cellular differences between schizophrenic and neurotypical individuals implicate difference in glutamate and GABA neurotransmitter systems [2], but it is unknown how cellular differences manifest as localized circuit dysfunction and how that leads to behavioral deficits.

**Fig 1:**
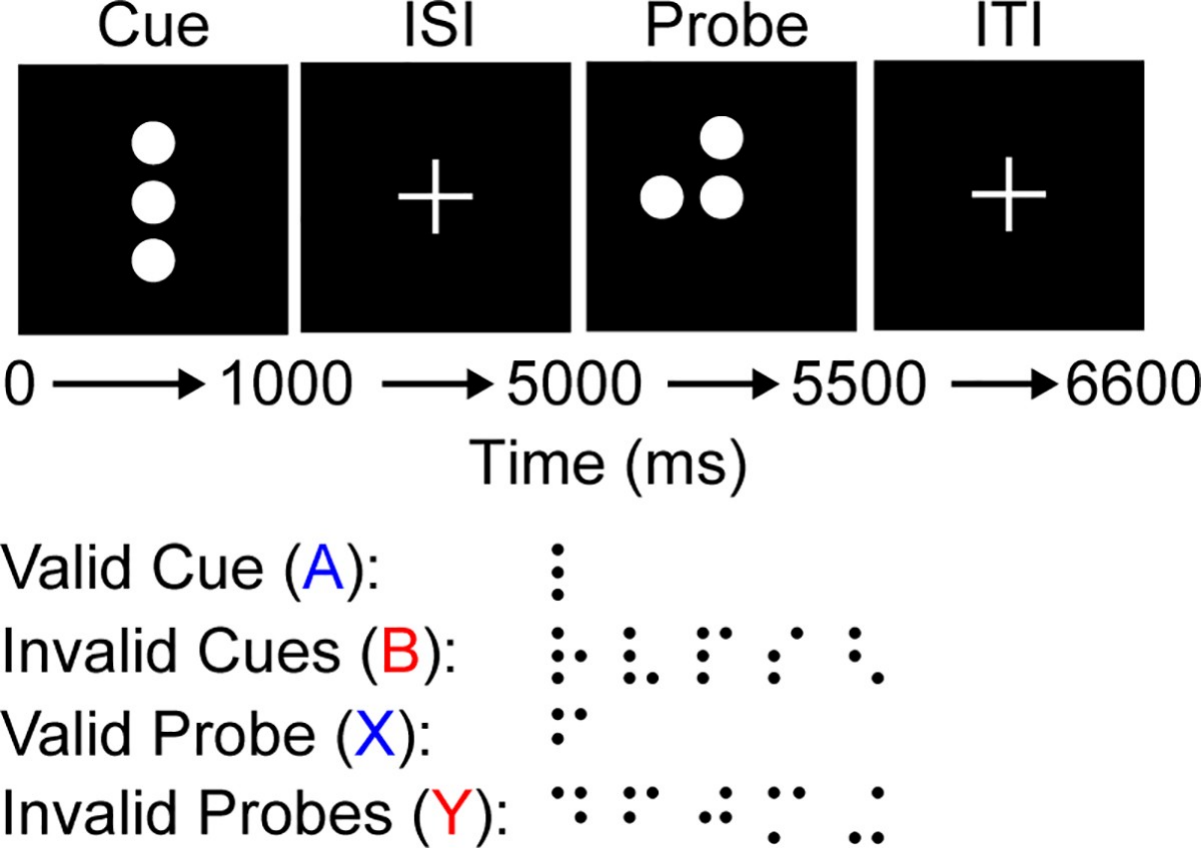
DPX task trials. Example of the DPX task as experienced by a participant. The participant observes various dot patterns during the cue and probe periods for 1 and 0.5 seconds, respectively. Between the cue and probe there is an interstimulus interval (ISI) of 4 seconds and after the probe an intertrial interval (ITI) of 1.1 seconds. The dot patterns can be grouped into A or B cues and X and Y probes. The A and X stimuli are considered ‘valid’ and, when observed sequentially (AX), the participant should make a left response; in all other cases the participant should makes a right response (i.e., for AY, BX, and BY combinations). There are a multiple B cues and Y probes, which can be used to assess the participants’ ability to differentiate them from the valid stimuli. Participants typically engage with 150 cue-probe trials that are 64% AX, 16% AY, 16% BX, and 4% BY. These are unevenly distributed to create a prepotent response towards the left (AX) option.

One common behavioral deficit among schizophrenic individuals is that they have difficulty with context integration [3–9], which is the process of combining currently available and previously observed information to determine an appropriate action [3,4,10,11]. The AX Continuous Performance Task (AX-CPT) [12] and its derivative the Dot Pattern Expectancy task (DPX) [13] are frequently used to differentiate between various context integration deficits. These tasks consist of a series of cue-probe pairings (Fig 1), with an interstimulus interval between the cue and probe. The response that the participant should perform depends upon the information provided by the cue and probe stimuli. The participant should only perform a left response when the cue and probe are both ‘valid’, and should make a right response for all other cases. There are a total of 6 cues and 6 probes, but only 1 cue and 1 probe are considered valid. The valid cue and probe are more frequently presented during the task and are typically referred to as the A cue and X probe. All other cues and probes, which are invalid, are referred to as B cues and Y probes. The probe presentation is followed by a brief intertrial interval (ITI). Participant errors on the various trial types indicate different deficits. Errors when an A cue is followed by an X probe (AX) indicates general difficulty with the task, AY errors indicate difficulty with inhibiting the prepotent response, and BX errors indicate working memory deficits.

Individuals with schizophrenia often exhibit higher error rates on AX and BX trials and longer reaction times when they engage with the AX-CPT and DPX tasks [4]. College students exhibited a similar pattern of increased AX and BX errors when they were administered ketamine [14], which is a noncompetitive NMDA antagonist and suggests that changing the underlying EI balance drives these behavioral changes. Rhesus macaques also exhibited more errors on BX trials and greater reaction times under NMDA antagonists ketamine [15] or phencyclidine [16]. This impairment of more frequent BX errors coincided with disruption of neural populations within the macaque’s prefrontal cortex (PFC) [16], which had been seen to maintain cue-information during the ISI [17]. Schizophrenic patients have exhibited reduced activation of the PFC during the ISI of the DPX task [3,6,18]. These results suggest that an EI imbalance in the PFC may underlie these context integration deficits but leave open the question of how systemic drugs [14–16] and global changes [2] specifically affect PFC function.

Glutamatergic and GABAergic neurotransmitter systems exist throughout the entire brain, so it is unclear why a global EI imbalance would result in a localized PFC dysfunction. A classic argument for the dynamics of working memory, a necessary component of context-integration, is that pyramidal cells and interneurons within the PFC create a persistent pattern of activity that maintains the representation of information over brief time periods [19]. While other models of memory maintenance exist [20,21], we chose to build on this classic model as it is known to be sensitive to EI balance [22–25]. When EI balance favors excitation, these models result in epileptic firing of pyramidal cells or are prone to representational drift, and when it excessively favors inhibition, the models are incapable of maintaining representation over time. This leaves a restricted parameter range in which working memory can function [25] and, thus, suggests a mechanism by which a global shift in the EI balance could result in the PFC becoming unable to maintain a representation of context over time. We suggest that the reason these systemic drugs affect PFC functionality more than other systems is that the EI balance in PFC is more sensitive to these global shifts than other networks that have different EI balances due to its unique function [26].

Context integration requires that actions depend on both current and previous information, and, thus, requires multiple interacting neural circuits to guide behavior. We hypothesized that some of the changes to EI balance would only result in dysfunction to our PFC analogue, and that this dysfunction would result in context integration deficits that were similar to the performance of schizophrenic participants on context-integration tasks. To test the viability of this hypothesis, we explored EI balances in a multi-structure agent attempting the AX-CPT/DPX task. One component of the agent had an EI balance that acted as a perceptual system, representing the current cue/probe input, while another component had an EI balance that acted as a memory system, maintaining the cue even when the probe was provided. Global manipulations affect these systems differently, reproducing the errors seen in global NMDA manipulations and in schizophrenic patients. A thorough exploration of the parameters of these networks revealed new insights into their interaction and suggest testable predictions for future experiments.

## Results

### Differentiating perceptual and memory networks

**Fig 2:**
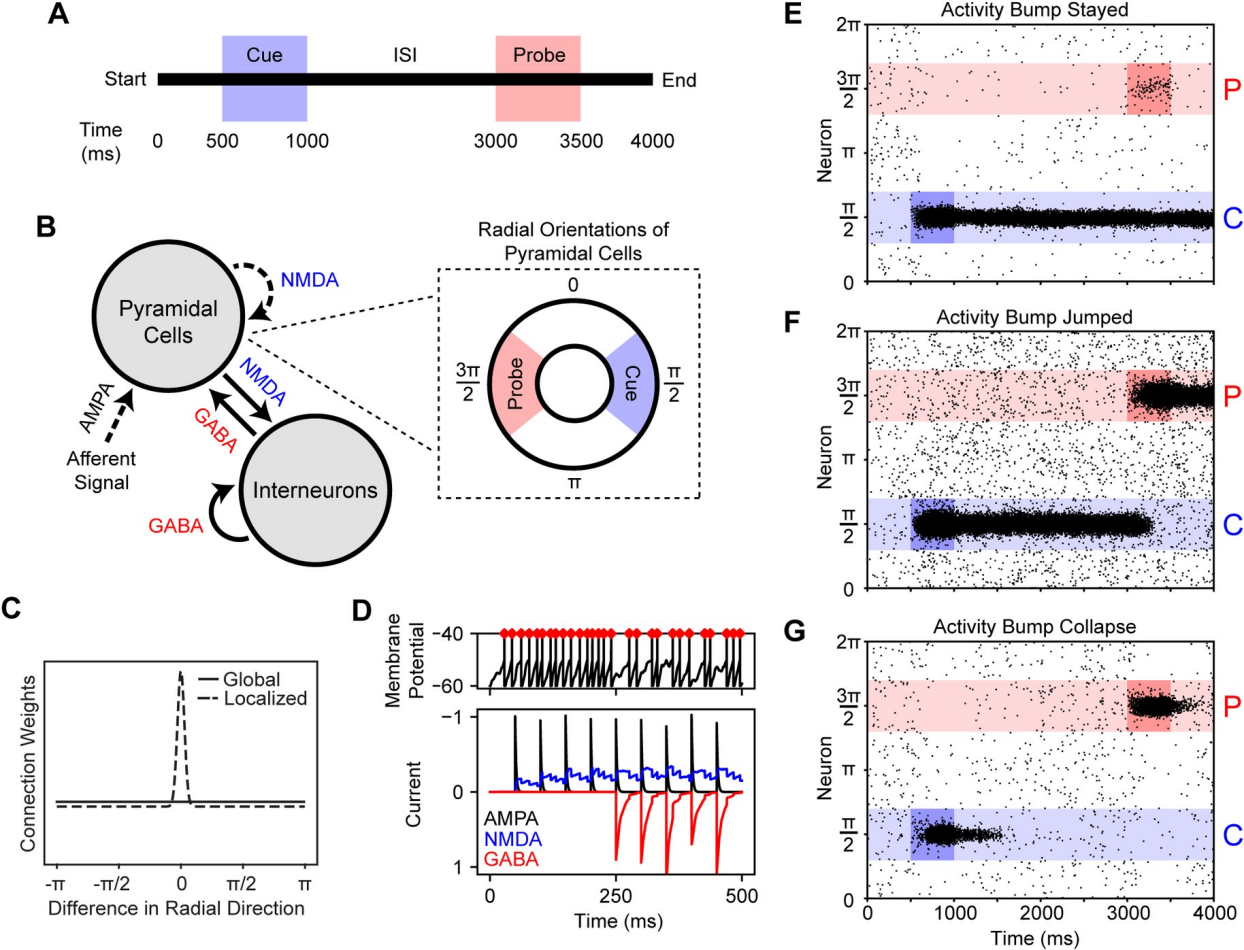
Simple cue-probe experiment and model design with example raster plots. **A.** Temporal structure of cue-probe trials. During each trial, the cue and probe were both presented for 500 ms at 500 and 3000 ms, respectively. **B.** Design of the ring attractor and important receptor subtypes for excitatory and inhibitory connections. The ring attractor consisted of 1024 pyramidal cells and 256 interneurons. The pyramidal cells were assigned radial directions and those near π/2 and 3π/2 were associated with the cue and probe stimuli, respectively. Connections indicated with a dashed line were spatially localized whereas those indicated with a solid line were not. **C.** Plot of the weight distributions of localized and global connections. Localized weights were more strongly connected to pyramidal neurons with a similar radial direction. The difference between global inhibitory and localized excitatory connections resulted in the creation of activity bump [23]. **D.** An example of AMPA, NMDA, and GABA receptor currents and how those manifested as spiking activity. In this example, EPSPs were induced at a rate of 20 hertz for the entire duration and IPSPs were induced at a rate of 20 hertz starting at 250 ms. The introduction of the IPSPs reduced the spiking rate (red diamonds) shown in the top panel as the excitatory and inhibitory currents interacted. Leak and noise currents are omitted. **E-G.** Example raster plots that show how activity bumps are induced and represent the cue and probe. **E.** With activity bump maintenance, the neural representation of the cue stayed at π/2 despite afferent signal at 3π/2 during the probe (NMDA_g,pyr_ = 0.37 μS, NMDA_g,int_ = 0.33 μS, AMPA_g,Aff_ = 0.9 μS). **F.** In the case of activity bump jumps, the neural representation switched from the cue to the probe (NMDA_g,pyr_ = 0.37 μS, NMDA_g,int_ = 0.30 μS, AMPA_g,Aff_ = 0.9 μS). **G.** The activity bump can also collapse prior to probe presentation (NMDA_g,pyr_ = 0.33 μS, NMDA_g,int_ = 0.30 μS, AMPA_g,Aff_ = 0.9 μS).

Perception and memory fulfill different roles in information processing and require different characteristics that make them differentially affected by EI balance. To explore what characteristics were required for these networks, we constructed a ring attractor (Fig 2B) that had a similar design to previous spiking neuron models of spatial working memory [23,24]. This ring attractor was stimulated at π/2 at 500 ms (cue) and at 3π/2 at 3000 ms (probe) (Fig 2A) and we observed how the network’s neural activity responded to these stimuli. We are not arguing that a ring attractor is necessary for simulating working memory, but we simply used this structure to craft balanced networks that were, essentially, categorical due to heavily localized intra-excitatory connections (Fig 2C). Individual neurons were affected by glutamate and GABA (Fig 2D), and the EI balance of this network was controlled by separately modifying the NMDA receptor conductances of excitatory and inhibitory cells.

**Fig 3:**
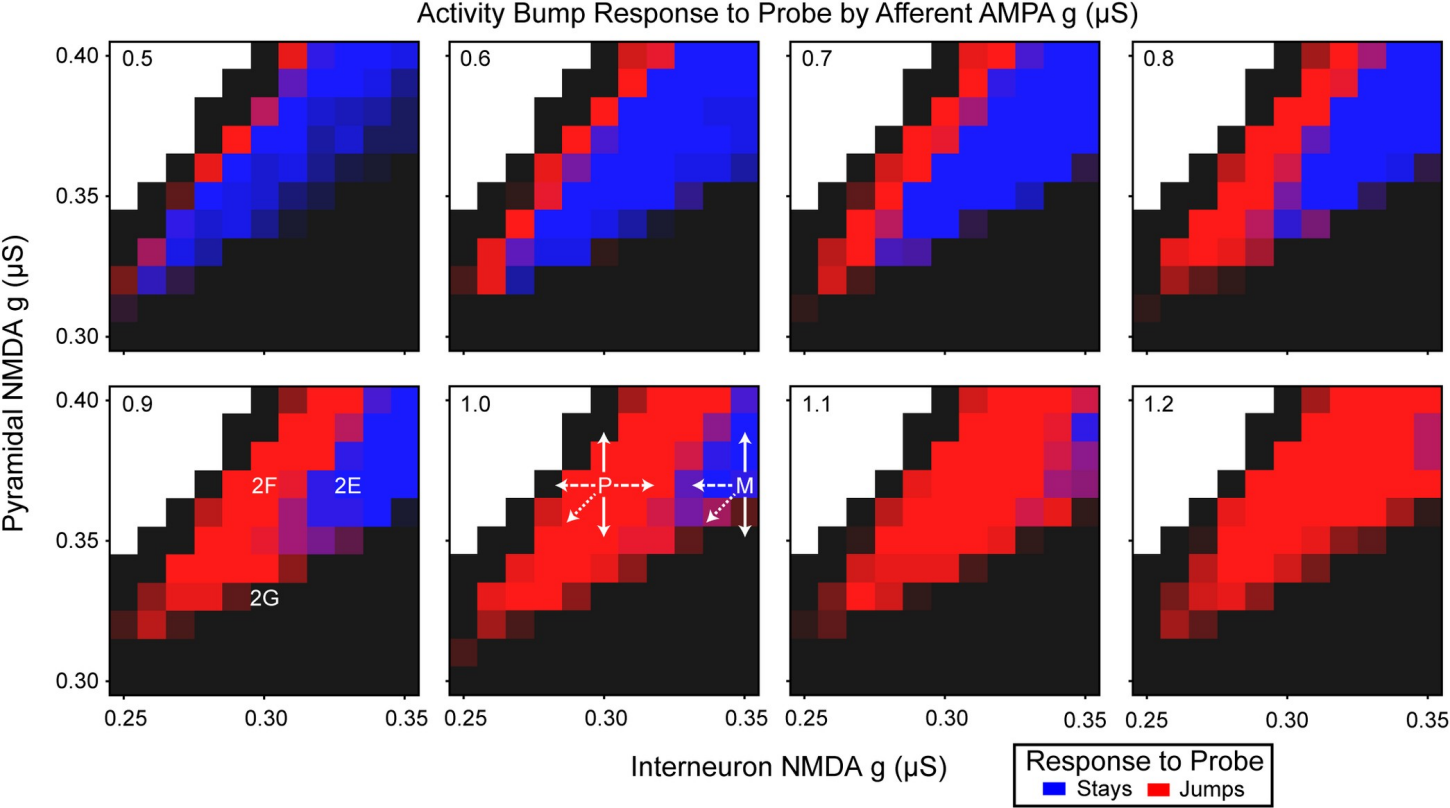
Activity bump response to the probe as a function of excitation, inhibition, and afferent signal. The afferent AMPA conductance (g) is indicated in the top left of each panel. The degree of blueness indicates the percentage of trials in which the activity bump maintained the cue representation, and redness indicates the percentage of trials in which the activity bump jumped to the probe representation. The black and white regions indicate excitation-inhibition balances that were incapable of maintaining representation of the cue, albeit for different reasons. The degree of blackness indicates the percentage of trials in which the activity bump collapsed prior to the probe or that the cue representation was never initiated, and white indicates the parameter region in which the pyramidal cell firing was epileptic (i.e., showed an extremely high firing rate across the entire population that does not represent information). The P and M in the 1.0 AMPA conductance panel are the locations of the context-integration agent’s perception and memory networks (Fig 4A) within NMDA conductance parameter space. The arrows coming from these indicate various manipulations that were performed to these networks. The solid line is pyramidal cell NMDA conductance manipulation, the dashed line is interneuron NMDA conductance manipulation, and the dotted line is manipulation of both.

We explored how the NMDA parameter space affected the ability to maintain activity bumps and how those activity bumps responded to afferent stimulation. We systematically varied the pyramidal cell NMDA receptor conductance, NMDA_g,pyr_, between 0.30 and 0.40 μS and the interneuron NMDA receptor conductance, NMDA_g,int_, between 0.25 and 0.35 μS by steps of 0.01 μS. These networks were then subjected to varying amplitudes of afferent signal by systematically assigning the afferent AMPA conductance, AMPA_g,Aff_, parameter between 0.5 and 1.5 μS in steps of 0.1 μS. The behavior of the networks could be classified into three meaningful categories: 1) the activity bump was formed and did not change with the probe (Fig 2E), 2) the activity bump tracked new stimuli (Fig 2F), or 3) the activity bump collapsed between the cue and probe stimuli (Fig 2G). The parameter combinations that gave rise to the displayed outcomes are shown in Fig 3‘s 0.9 μS AMPA g panel.

Networks with greater NMDA receptor conductances had greater inertia (Fig 3). The majority of networks in the explored parameter range were incapable of sustaining an activity bump because either the activity bump failed to form or quickly collapsed (black) or an activity bump could not be maintained because the network was epileptically firing (white). Between these two regions the network functionally tracked stimuli (red), like perception, or retained the original stimulus (blue), like working memory. As the interneuron and pyramidal cell NMDA receptor conductances simultaneously increased, the network’s inertia increased. High-inertia working-memory ring attractors are those that have similar NMDA receptor conductances for interneurons and pyramidal cells and both are relatively high. Low-inertia perception-like networks have relatively greater pyramidal NMDA conductance, but not to the point that these networks are incapable of representing the stimuli due to epileptic firing. These simulations show that the EI imbalances seen in more abstract population firing-rate models [25,27] also appear in biophysically-realistic models that take NMDA, GABA, and AMPA dynamics into account, which allowed us to explore the consequences of NMDA manipulations on these networks.

### A Context-integration agent

**Fig 4:**
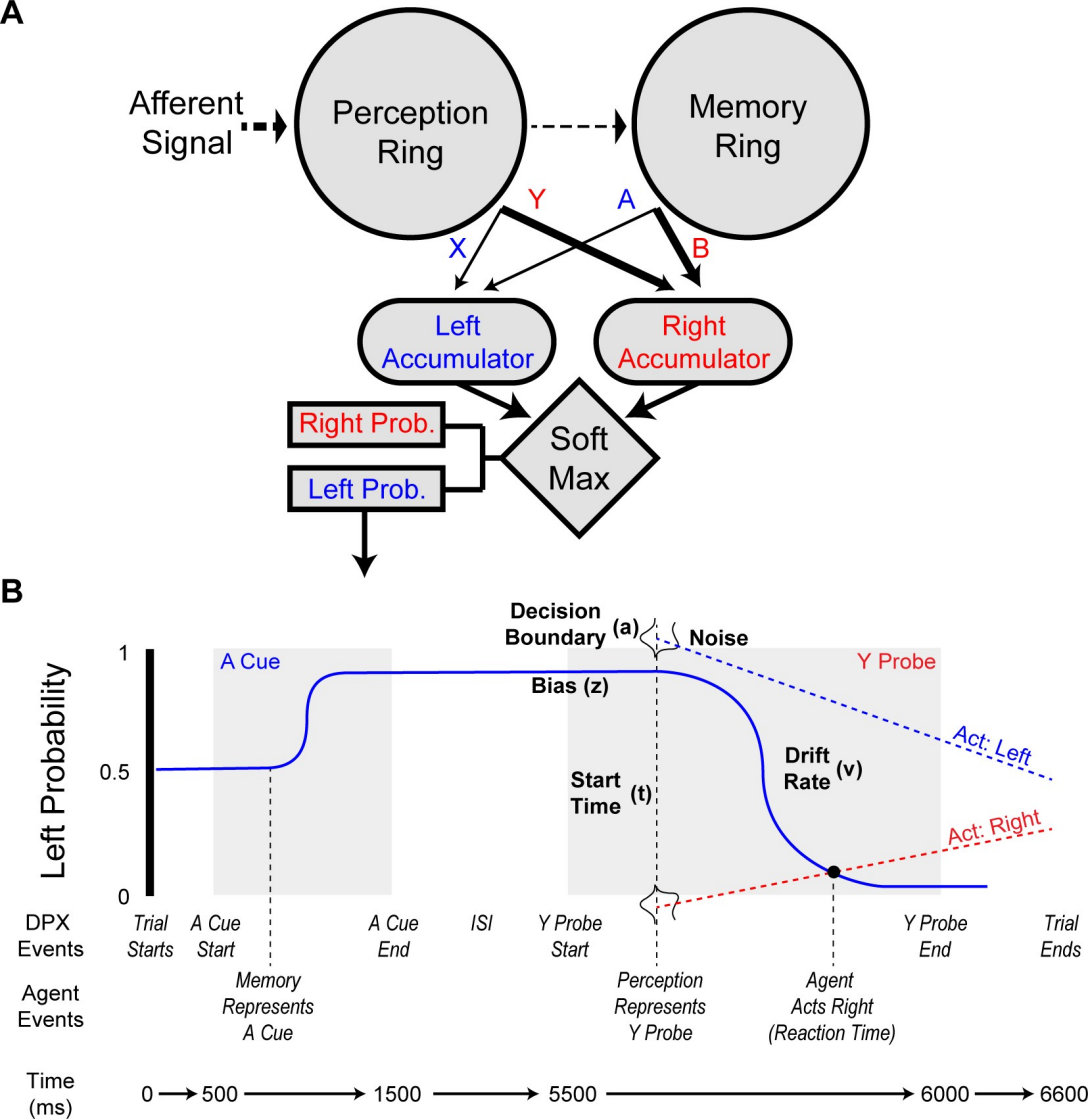
DPX task and agent design. **A.** Design of the DPX agent. The agent was comprised of perception and memory ring attractors that represented the current stimuli and the context, respectively (due to their underlying EI balances). Both ring attractors were structurally identical to the one specified in Fig 2B. The perceptual network’s pyramidal outputs projected weakly to the memory ring, providing its representational inputs. As with the ring attractor in Fig 2B, certain areas of the ring represented the cue and probe stimuli. How well the cue and probes were represented in the networks was used to inform the agent’s decision making process. Pyramidal cell activity in the ring attractors projected to leaky left and right response accumulators that utilized a softmax decision making algorithm to determine the agent’s likely response to the probe. Connection weights to the left accumulator (i.e., A from the memory ring and X from the perception ring) are weaker than those to the right accumulator (i.e., B from the memory and Y from the perception ring), because the representations of B and Y needed to override the A and X representations during decision making. **B.** Temporal structure of an AY DPX trial and how the agent’s decision making process changed in response to task events. The gray regions indicate when the cue and probe were presented (not to temporal scale). As the agent represented information about the cue and probe, its likelihood of engaging in a left or right response changed. For example, representation of the A cue caused the agent to be much more likely to engage in a left action, but as the Y probe information was represented, the agent became unlikely to engage in a left action. A drift-diffusion-like decision process was used to determine the timing and choice of the agent’s actions. As soon as the agent’s perception network represented the probe (X or Y), it initiated its decision making process. When a collapsing left or right response boundary crossed the left action probability, the agent engaged in the associated action at that time. This decision making process did not formally specify drift-diffusion-model-like dynamics, with the exception of the decision boundaries (*a*). However, the decision making process’ created similar constructs. How the components of this process are akin to an evidence accumulation process are identified with the terms and parameters (indicated *a*, *t*, *v*, and *z*) that are commonly used in the DDM literature.

**Fig 5:**
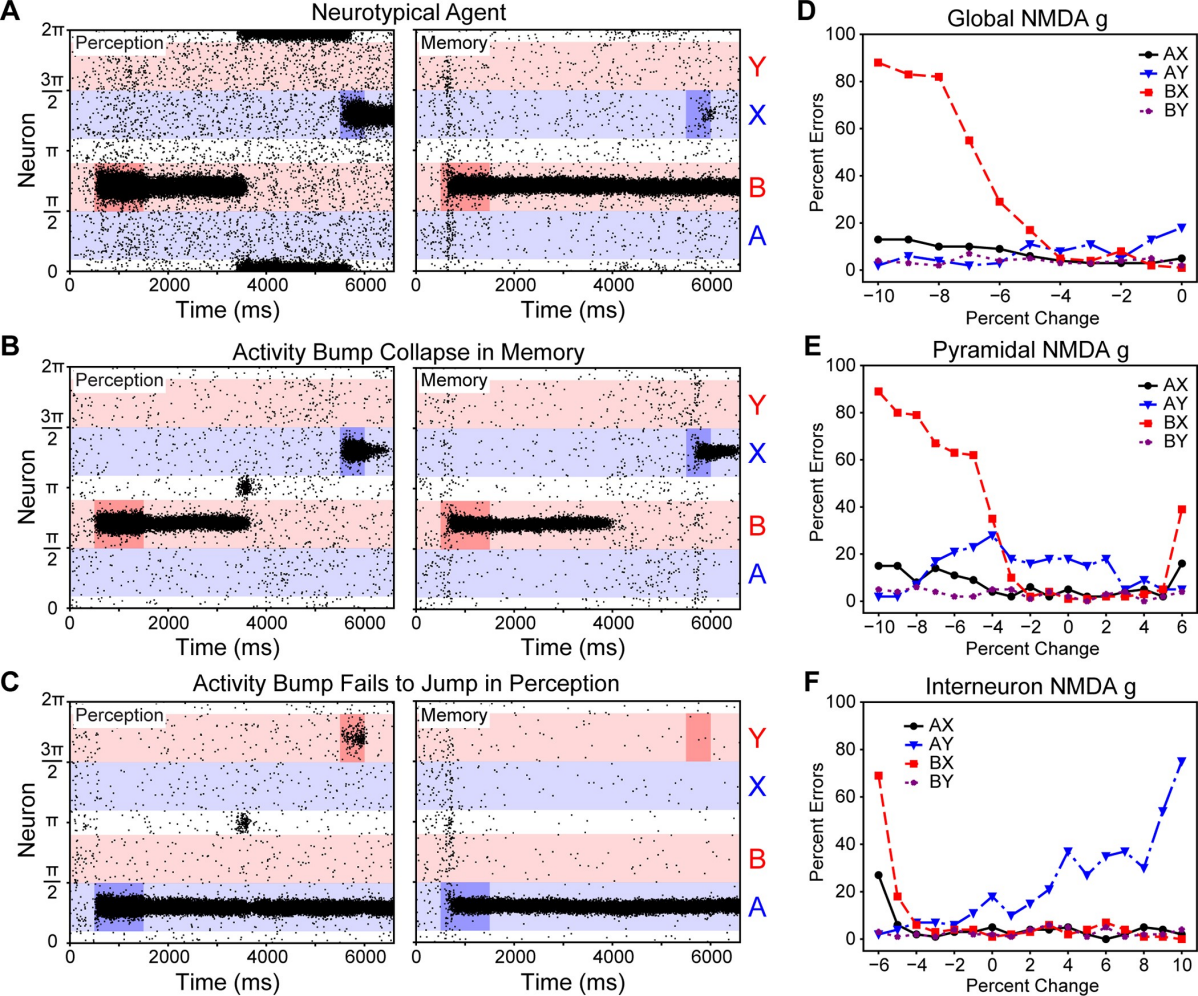
DPX task performance of neurotypical and divergent agents. **A.** Raster plots of a neurotypical agent’s pyramidal cell firing, which successfully maintained representations of the ‘B’ cue and ‘X’ probe (Perception: NMDA_g,pyr_ = 0.37 μS, NMDA_g,int_ = 0.30 μS; Memory: NMDA_g,pyr_ = 0.37 μS, NMDA_g,int_ = 0.35 μS). **B.** Raster plots of a trial in which the activity bump representation in the memory network collapsed during the ISI (-8%—Perception: NMDA_g,pyr_ = 0.3404 μS, NMDA_g,int_ = 0.30 μS; Memory: NMDA_g,pyr_ = 0.3404 μS, NMDA_g,int_ = 0.35 μS). **C.** Raster plot of a trial in which the activity bump representation in the perception network fails to track current stimuli (+8%—Perception: NMDA_g,pyr_ = 0.37 μS, NMDA_g,int_ = 0.324 μS; Memory: NMDA_g,pyr_ = 0.37 μS, NMDA_g,int_ = 0.378 μS). **D.** How an exogenous NMDA antagonist, like ketamine, may affect the rate of DPX task errors. **E.** Increasing error rates of the different trial types as the pyramidal NMDA g was systematically changed. **F.** Increasing error rates of the different trial types as the interneuron NMDA g was systematically altered.

**Fig 6:**
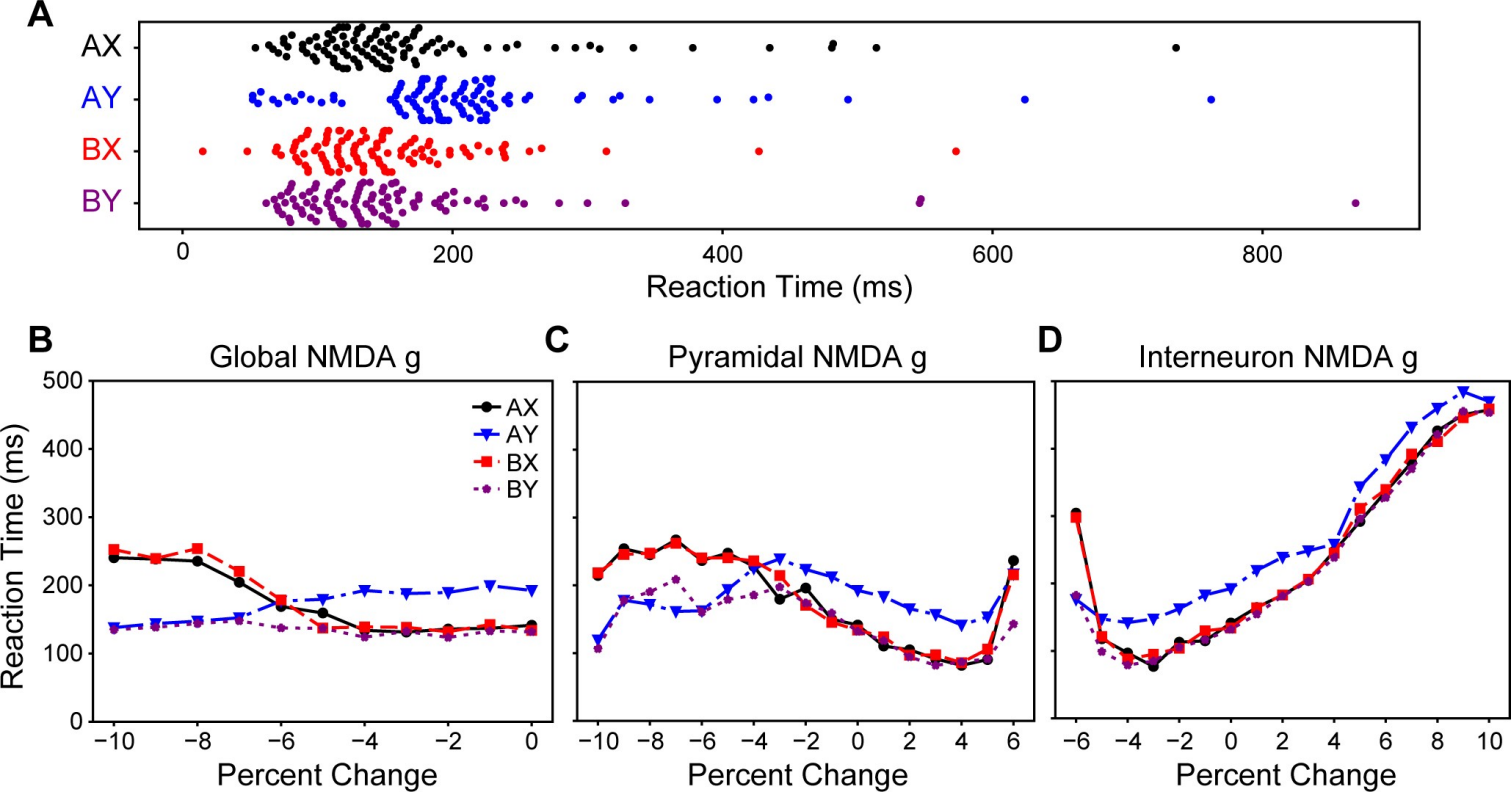
EI balance affects the agent’s reaction times. **A.** Swarm plots of the neurotypical agent’s reaction times during the four trial types. As is in the DPX task literature, there was a delayed response on AY trials. **B.** Reaction times bifurcated as the NMDA conductance of all cells was reduced. The reaction times separated by the probe. **C.** Reaction times also bifurcated as the pyramidal cell NMDA conductance was reduced, but increased across all trial types as it approached a 6% increase in pyramidal NMDA conductance. **D.** Reaction times of all trials increased as the NMDA conductance on interneurons increased, and also increased as it reached a 6% reduction.

In order to examine how systemic NMDA changes on these different networks could affect behavior, we constructed an agent (Fig 4A) with perception and memory networks (P and M in Fig 3, respectively) such that the interaction of these networks was capable of solving the DPX task. These networks filled roles within the agent that are analogous to that of the posterior parietal cortex (PPC) and prefrontal cortex (PFC), respectively [25]. The agent was comprised of two ring attractors in a feed-forward architecture and the activity of each ring attractor informed a Softmax decision-making process. This agent was designed to engage with the DPX, which we slightly modified by adding a distractor at 3500 ms to ensure that activity in the perception module was not maintaining the memory module’s activity during the ISI. The network showed similar properties in the absence of a distractor, but was less sensitive to NMDA conductance changes (S1 Fig), so we included a distractor in the simulations to provide a more thorough exploration of the perceptual and memory networks. The perception and memory ring attractors each had 5 competitive memory states (A, B, X, Y, and other), and these states determined whether the agent was more likely to make a left or right response. When the agent’s perception network began representing the probe, it cued a decision making process (Fig 4B) that determined the agent’s action and reaction time. Briefly, this decision making system was analogous to a drift-diffusion model (DDM) [28,29] and used the agent’s determination of the likely correct response to create features akin to a DDM’s response bias (z), drift rate (v), and starting time (t) parameters. Decision making components that were not determined by the agent, the decision boundary (a) and noise, were used to create independent collapsing response thresholds. The agent’s reaction time was determined from when the response probability crossed one of the collapsing thresholds. Full details on the model can be found in the Methods. The neurotypical (default) agent properly represented information about the cue and probe in its memory and perception networks (Fig 5A) and appropriately used that information to determine its actions. The agent was designed to have a slight propensity to make errors on AY trials, because this is commonly observed when neurotypical humans engage with the AX-CPT and DPX tasks [4]. The distributions of the neurotypical agent’s reaction times (Fig 6A) showed a similar pattern to that seen in a neurotypical human’s performance, including that the median reaction time on AY trials tended to be longer than on other trials. The reaction times only included the decision-making process and did not include perceptual processing prior to the perceptual network, nor did it include motor action processes.

Adjusting the agent’s NMDA conductance parameters produced schizophrenic-like context integration deficit errors. We systematically and simultaneously varied the NMDA receptor conductances of pyramidal cells, interneurons, or both in the perception and memory networks (Fig 5D, 5E, and 5F). There were multiple causes of increased AX and BX error rates (Fig 5D, 5E, and 5F), which are similar to those exhibited by schizophrenic patients on the DPX task [4,5]. Increased AX and BX error rates were produced by the reduction of NMDA receptor conductances of pyramidal cells (Fig 5E), interneurons (Fig 5F), or both (Fig 5D) or by the increase in pyramidal cell NMDA receptor conductance (Fig 5E). AX and BX errors were caused when the activity bump collapsed wihin the memory ring attractor during the ISI (e.g., Fig 5B). When information about the cue was lost during the ISI, the agent lacked that information when responding to the probe and, thus, based its actions solely upon the available probe information.

The increase in AX and BX error rates coincided with changes in the agent’s reaction times, and these changes could be separated into two patterns. As the global or pyramidal cell NMDA conductances were reduced (Fig 5D and 5E) reaction times bifurcated by the probe that was presented (Fig 6B and 6C). In contrast, as the pyramidal cell NMDA receptor conductance was increased (Fig 5E) or the interneuron NMDA receptor conductance was reduced (Fig 5F) there was a more general increase in the reaction times (Fig 6C and 6D). These two patterns, respectively, coincided with whether the network’s EI ratio became more inhibitory (downward right of Fig 3) or more excitatory (upper left section of Fig 3). Individuals with schizophrenia tend to have delayed reaction times, relative to neurotypical individuals, that are similar across all trial types [4,5]; the agents with more excitatory EI ratios tended to produce this pattern of behavior and were most like that of individuals with schizophrenia. That a bifurcation reaction-time pattern is produced when global NMDA conductance is reduced–like an NMDA antagonist drug manipulation–is interesting because a similar pattern has been observed when a pair of macaques were administered an NMDA antagonist [15].

Context integration has not been studied in individuals with autism spectrum disorder, but one pattern of errors that our agent produced suggested some potential utility for that application because it created fixation and delayed responding. EI imbalances created more AY errors when the interneuron NMDA conductance was increased (Fig 5F). This increase in AY errors was due to the perception ring attractor’s activity bump fixating on the probe stimulus (Fig 5C). This qualitative difference can also be seen when examining the corresponding median reaction times (Fig 6D). The agent’s perception network begins to fixate as its NMDA conductance makes it cross the perception-memory threshold (P in Fig 3 moving right).

### EI Balance and tuning curves

**Fig 7:**
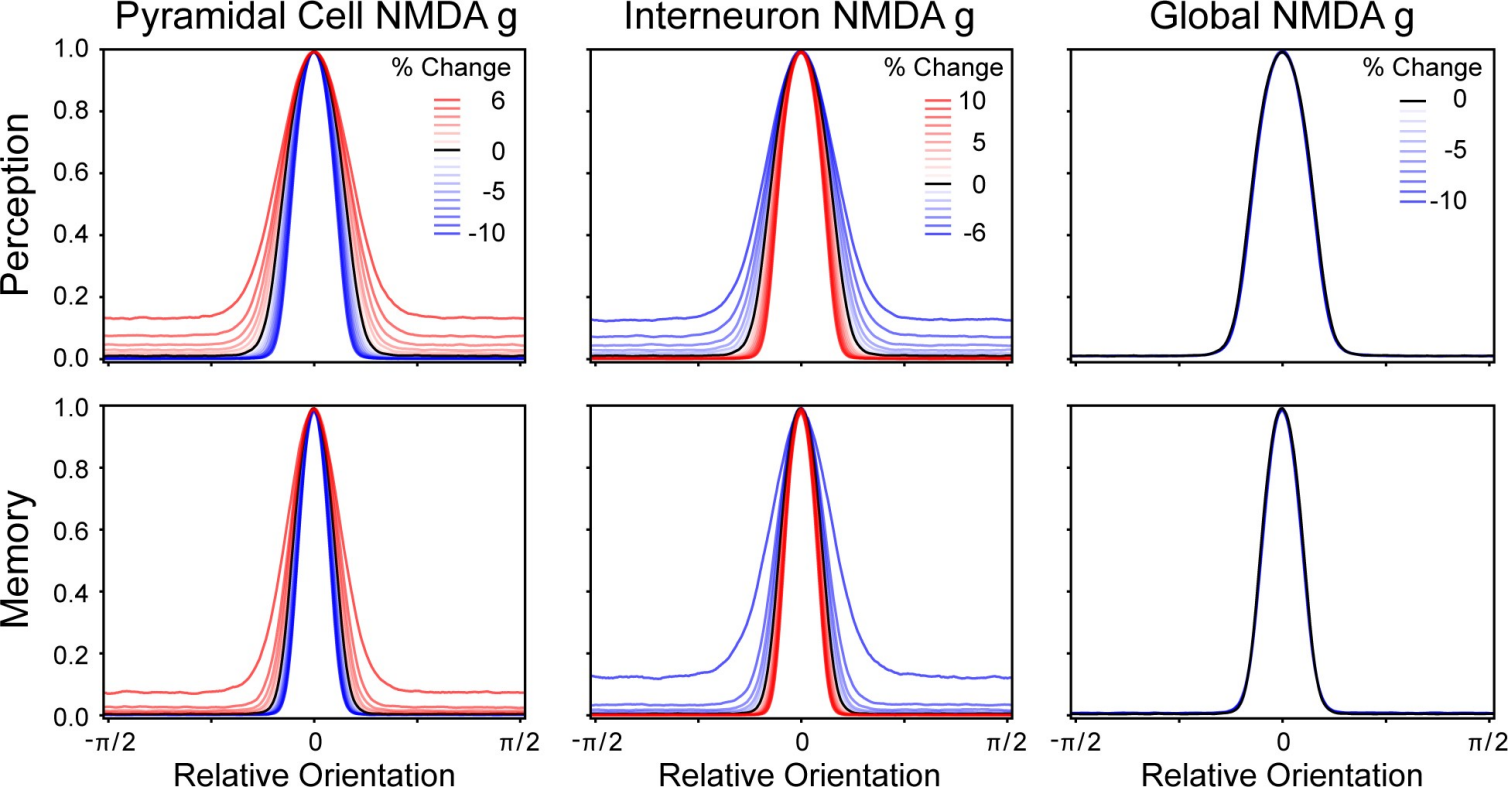
EI balance affects tuning curve shapes. As NMDA receptor conductance of the pyramidal cells and interneurons were systematically manipulated, it resulted in the representational tuning curves changing in width. As the NMDA conductance manipulation approached the epileptic corner of network firing (top left in Fig 3), the tuning curves became wider, and as the networks became unable to initiate or maintain a representation (bottom right in Fig 3), the tuning curves became more narrow. Notably, as the NMDA conductance parameters were globally changed (moving along the bottom left to top right diagonal in Fig 3), there was no change in the tuning curve’s shape.

The potential utility of viewing EI balance as an intertwined negative-feedback loop is underscored by how the balance affects the representation of information. As the EI balance was altered, the tuning curve representation of information also changed (Fig 7). When the network’s EI balance more strongly favored excitation (i.e., greater pyramidal cell NMDA conductance or less interneuron NMDA conductance) the tuning curves became wider with a higher base. The corollary of this was also true; the tuning curves became narrow when the EI balance favored inhibition (i.e., reduced pyramidal cell NMDA conductance or greater interneuron NMDA conductance). A global change in NMDA conductance, however, had no effect upon the tuning curve shape because it proportionately affected all cells and thus failed to change the EI balance. While changing the NMDA conductance of pyramidal cells and interneurons separately affected the excitation and inhibition components of that balance, their net effect on tuning curve shape was quite similar. Memory and perception networks differed in their tuning curves due to their differences in EI balance, which were required for those network’s to fulfill their function. This suggests EI balance changes could produce measurable differences in the proportions of active neurons in different networks.

### Localizing the source of context-integration errors

**Fig 8:**
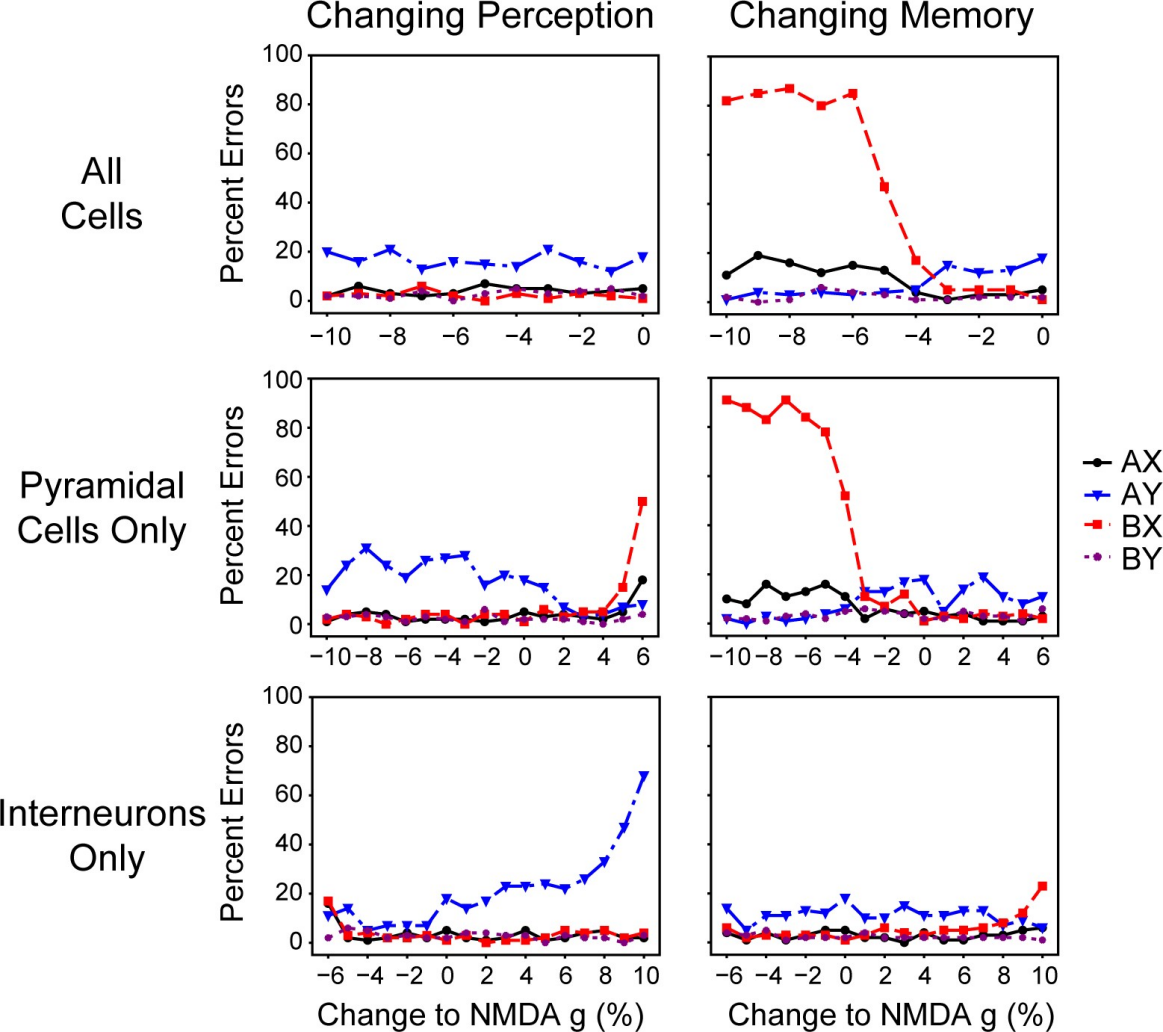
Specific network manipulations caused mistakes on various trial types. It is possible to determine whether the perception or memory network caused the agent to make an error by systematically altering the NMDA conductances of pyramidal cells and interneurons within either the perception or memory networks. Errors on AX and BX trials were most easily caused by the memory network when it began to have difficulty maintaining the representation of the cue during the ISI (middle and top right panels). Manipulations of the perception network also caused AX and BX errors (middle left panel), but this was due to it causing consequential changes in the memory network’s functioning (Fig 10). Errors on AY trials increased as the perception network became more inhibitory by either reducing pyramidal cell NMDA conductance or increasing interneuron NMDA conductance (middle and bottom left panels).

Global changes in NMDA receptor conductances affected the entire agent, but created localized dysfunction. Assuming that the agent’s perception and memory networks are truly analogous to the PPC and PFC, we would expect that the source of BX errors in DPX decision making to lie within the memory network because the PFC of schizophrenic participants is hypofunctional during the ISI of the DPX task [3,6,18]. We assessed this hypothesis by systematically manipulating either the memory or perception network’s NMDA receptor conductances (Fig 8).

**Fig 9:**
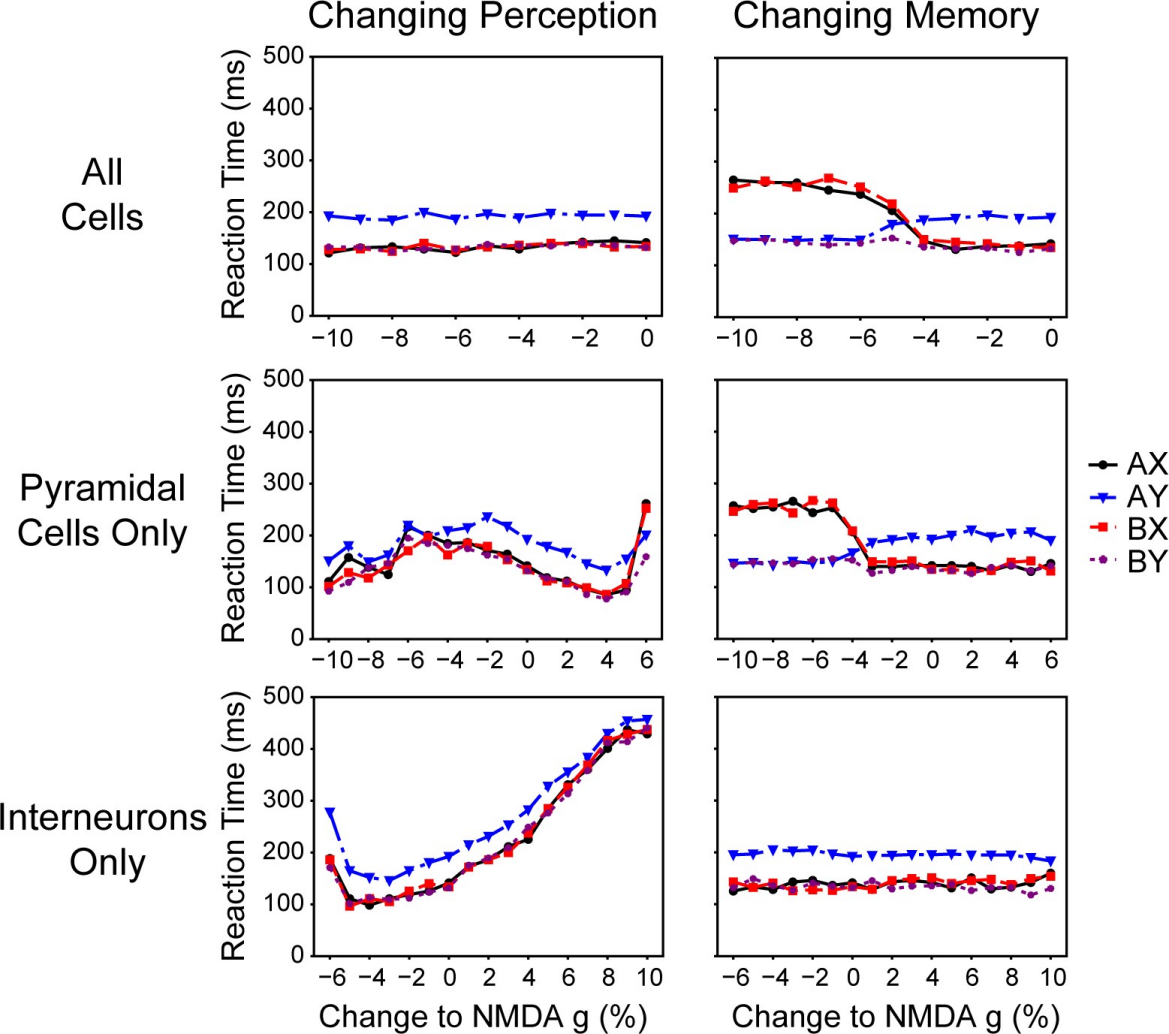
Specific network manipulation affected reaction times. The bifurcation of reaction times was specific to when the memory network was the direct cause of errors on AX and BX trials (top and middle left panels). Overall changes in reaction times were primarily caused by the perception network (middle and bottom left panels). This was due to the perception network becoming noisy (right and left of the middle and bottom left panels, respectively) or beginning to fixate (left and right of the middle and bottom left panels, respectively).

**Fig 10:**
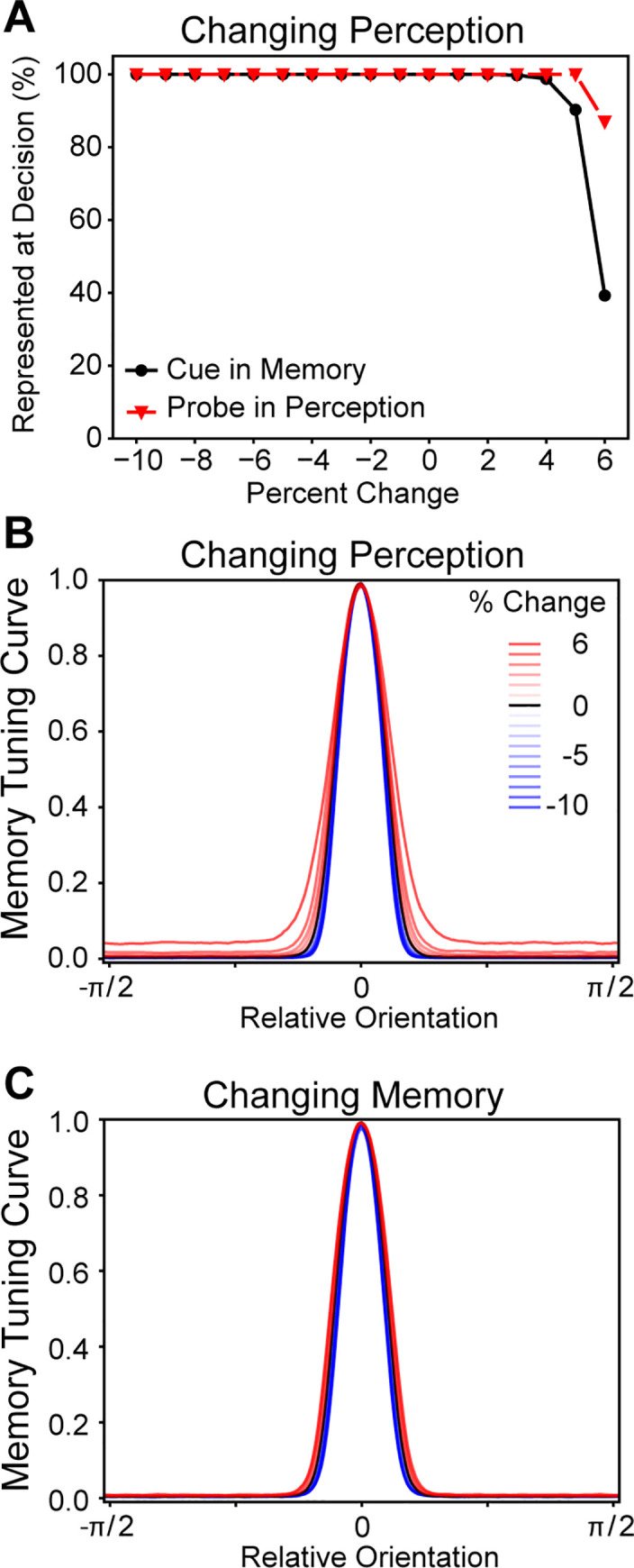
Changing the perception network’s pyramidal cell NMDA conductance caused memory malfunction. **A.** As the perception network was altered it caused the memory network to lose its representation of the cue, but had little effect on the perception network representing the probe. **B.** As the perception network’s pyramidal cell NMDA conductance increased, it caused the memory network’s tuning curve to become wider. **C.** The memory network’s tuning curve was barely affected by its own pyramidal cell NMDA conductance being changed.

This experiment confirmed that the memory component was the source of errors on the AX and BX trials, but in a complicated fashion. Dysfunctional memory was the simple cause of significant AX and BX errors when that network’s pyramidal cell NMDA conductance or global NMDA conductance was reduced (Fig 8), but this also caused a bifurcation in reaction times (Fig 9). Increasing excitation in the perception network caused greater AX and BX errors (Fig 8), but did this indirectly by making the memory network lose representation of the cue (Fig 10A). Increasing the perception network’s pyramidal cell NMDA conductance strongly affected the memory network’s tuning curve (Fig 10B), but altering the memory network in the same way had very little of an effect (Fig 10C). At high pyramidal cell NMDA conductances the perception network was able to fulfill its function of representing current information, but its noisiness and higher firing rate caused the memory network to no longer be able to fulfill its function (Fig 10A). This suggests that there may be two sources of errors in patients who are unable to solve the DPX task–memory deficit errors that produce a bifurcation in reaction times, and perceptual deficits that cause memory deficits through a secondary consequence that does not produce that bifurcation in reaction times. These different underlying deficits may require different treatment paradigms.

## Discussion

The alteration of EI balance within our agent resulted in many of the behavioral deficits that are observed among individuals with schizophrenia on the DPX task. The agent’s behavioral deficits arose from global changes in EI balances causing network-specific dysfunctions. The agent produced high error rates on BX trials, like those exhibited by individuals with schizophrenia [4,5], when the EI balance resulted in memory networks that were unable to consistently maintain representation of cue information over time. Similarly, the agent produced more errors on AY trials when the perception network’s inertia increased to the extent that it was resistant to changing its representation when exposed to new stimuli.

High rates of BX errors on the DPX was a common result of manipulating our agent’s EI balance, and that increase in errors was due to memory networks failing to maintain a representation of the cue. However, the reaction time data produced by the model helped identify which EI balance changes were most similar to those exhibited by individuals with schizophrenia. The higher error rates and slower reaction times on the DPX task exhibited by schizophrenic individuals seem to be most similar to when our agent’s EI ratio favored excitation (Figs 5E, 5F, 6C, and 6D). As the EI balance favors excitation, the tuning curves of the memory network become wider and elevated (Fig 7 memory network panels). This change in the tuning curves is a product of the EI balance and indicates how interneurons are only weakly constraining a wide recruitment of pyramidal cells. That weak constraint causes more noise in the representation of information. This noise reduced the rate of evidence accumulation in our model and, thus, slowed the reaction times. BX errors that were produced via other mechanisms tended to have clear representations of memory and, thus, still had relatively quick reaction times.

Cohen and colleagues have emphasized the potential role of dopamine within their model as a cause for context errors [6,19]. Our model produced similar effects to what they assumed would be due to dopamine, which suggests a conceptual degree of similarity between our modelling and theirs. Other models have suggested a role for dopamine changing EI balance dynamically [30,31]. Cohen and Servan-Schreiber put forward the argument that reducing the gain parameter of the neurons within a connectionist network was analogous to the effects of reduced dopamine and that it caused context loss [11]. Our manipulation of EI balance produced a similar effect via the increase in excitation through an emergent property of the network dynamics (Fig 7) despite no change in the network’s underlying structure. Conceptually, our model and theirs both increased noise within the memory networks despite utilizing entirely different mechanisms. In a later model, Braver, Barch, and Cohen made the argument that dopamine modulated the gating mechanism of afferent stimulation [19]. Our global NMDA conductance manipulation resulted in a similar reduction in the memory network’s inertia to change. Interestingly, it did not produce a similar effect in the perceptual network. In both models, these manipulations make the memory network more likely to track new stimuli, producing the observed behavioral deficits.

A computational interpretation of our model is that anything that disrupts the maintenance of cue representation during the ISI results in BX errors on the DPX task. Longer reaction times are produced when there is greater noise in the representation of information. The current behavioral data of schizophrenic individuals on context-integration tasks suggests that both of these are occurring and that there are multiple mechanisms that can produce this pattern. In our simulations we found that local consequences on EI imbalances via global NMDA conductance manipulations can produce this result. Besides NMDA conductance, EI imbalances can potentially be produced by numerous mechanisms. While it is possible to experiment with all of these mechanisms, the context-integration deficits seen in schizophrenia can be most clearly summarized and understood at the computational level as a poor ability to represent information over time due to noise causing instability.

### Model predictions

A frequent discussion within the clinical AX-CPT and DPX literature is how long the interstimulus interval needs to be in order to best assess context integration [4,9]. This debate has largely been a matter of balancing accuracy and specificity against the duration of testing. The underlying assumption of this debate is that a longer ISI permits the representation to decay to a larger degree that in turn leads to behavioral deficits. A different understanding of working memory is how well it can maintain a representation despite distractors, and that may prove to be a better approach to balancing these competing concerns. Within our model, changing the EI balance strongly affected the network’s inertia to change which made it more likely to track irrelevant stimuli. Our model suggests that a quicker test of an individual’s working memory would, thus, be the addition of distractors during the ISI and systematically changing them over the course of the experiment. This would permit a researcher to better assess the stability and excitability of an individual’s perception and working-memory circuits.

Macaques who have been administered NMDA antagonists while engaging with the DPX task [15,16] may utilize a different decision making strategy than humans. In studies with humans with schizophrenia, the higher error rates on BX trials coincides with a minor increase in errors on AX trials [4]; in contrast, macaques that were administered NMDA antagonists showed increased BX error rates, but no observable changes on AX error rates [15,16]. Due to the joint higher incidence rates of AX and BX errors in humans, we designed our agent to respond stochastically during a memory failure with a preference for target responses. A byproduct of this process is that when the representation lacked noise but still failed to maintain a memory representation, our agent tended to show a bifurcation in the reaction times with Y-probe trial responses being faster than X-probe trial responses. Blackman et al also observed a bifurcation in the reaction times of both macaques as the dose of ketamine increased but trials with X-probes were slower than those with Y-probes [15], which was opposite to the reaction times our agent produced. Rather than the stochastic process that we built our agent to use, the macaque’s decision making process could be heavily biased towards a left response which was only overridden when it was countermanded by B-cue or Y-probe information. Evidence supporting this hypothesis is that the macaques showed no change in AX error rates when the number of BX errors increased with ketamine administration [15], reaction times on X probe trials were faster than on Y probe trials when ketamine was administered [15], and more neurons were dedicated to maintaining a steady representation of the B cues than A cues [17]. When college students were administered ketamine while performing the AX-CPT task it resulted in simultaneous increases of AX and BX errors [14], unlike the macaques. Our agent was designed to engage in more stochastic responding to when it lacked cue information because that was a better description of human behavior, which caused increases in AX and BX trial errors. A strong bias towards the target response could explain why our stochastic model produced a similar bifurcation in response times to the macaques, but with slower reaction times on trials with X probes than on Y probes. This potential difference in decision making on similar context-integration tasks could be due to the training differences; the macaques were extensively trained on the DPX task [15,16] unlike the college students [14].

Our modelling indicates that it may be possible to parse two separate causes of BX errors on the DPX task by analyzing the reaction times and that important information can be gleaned via a drift diffusion model analysis of that data. If the reaction times of the trials with X probes are differentiable from trials with Y probes (the bifurcations in Fig 7B and 7C), then this suggests that the underlying process is losing information about the cue during the ISI, but that the representation of information is not particularly noisy. A drift diffusion model applied to participant reaction time data on the DPX task in this case should reveal lower decision boundaries (*a*) relative to controls. In contrast, if there are relatively longer delays in the reaction time across all trial types then it is likely that the memory representation is being lost during the ISI and that the representation of information is noisy. A drift diffusion model analysis of reaction times in this situation should have greater decision boundaries than controls. In both of the previous two cases, if the evidence accumulation rates caused by the X and Y probes are the same (unlikely since the X probe only provides useful information given a complete absence of cue information), then there would be no bias (*z*) towards left or right responses. Whether there is a difference in the evidence accumulation rates could be assessed by performing separate analyses of the X and Y probe trials and seeing if there is a difference in the accumulation rate parameter *v*. Examining the response initiation (*t*) parameter is also informative. Relatively smaller *t* parameter values indicate that the underlying circuits favor excitation or that afferent stimulation to the perception network is particularly strong. These hypotheses suggest that a drift diffusion model analysis of reaction times on the DPX could inform our knowledge of the underlying circuits that influence decision making and that there should be caution when grouping individuals with elevated BX errors since there can be different underlying processes that give rise to errors on those trials.

Our model suggests that context-integration tasks may also be usefully applied to autism spectrum disorders. It has been previously suggested that EI imbalances may have a role in autism spectrum disorder [2]. Perseveration on ideas and hyporeactivity to stimuli are symptoms of autism spectrum disorder and within the context of the DPX task these symptoms could result in errors on AY trials. Our model produced AY errors when the perception network began to function more like a memory network. Within our model, perception networks lost their tracking function when their EI balance became more inhibitory. This increased inhibition also had a profound effect on the agent’s reaction time, because the perception network required more stimulation before it would track new stimuli. This would manifest as later start times for an evidence integration process (t) in a drift diffusion model. This later initiation of evidence-integration has been observed in individuals with autism spectrum disorder when they engaged with a perception task [32] but not with a more socially relevant task [33]. These results suggest that it may be beneficial to investigate context integration among individuals with autism spectrum disorder.

### Summary

Alteration in EI balance resulted in our context-integration agent producing numerous behavioral deficits in line with some mental health disorders. Global shifts in the EI balance did not create overall problems, but rather regionally specific ones. This regional specificity was a result of increased susceptibility to EI manipulations, because our networks filled specific roles in information processing engendered by local neural characteristics. The regional dysfunctions in our model also created specific patterns of behavioral deficits that were consistent with those observed in schizophrenic individuals, autistic individuals, as well as humans and macaques receiving NMDA antagonists.

## Methods

### Leaky integrate and fire neurons

Pyramidal cells and interneurons were modeled as leaky integrate and fire neurons [34]. The change in their voltage potentials, *V*_*m*_, over time, *t*, were modeled as

$$C_{m}\frac{{dV}_{m}}{dt}=-I_{NMDA}-I_{GABA}-I_{Leak}-I_{Noise}-I_{Aff}$$

where *C*_*m*_ is the membrane capacitance, I_NMDA_ is the current from NMDA receptors, I_GABA_ is the current from GABA receptors, I_Leak_ is the leaky membrane current, I_Noise_ is current from task-irrelevant excitation, and I_Aff_ is the current from afferent signal. All cellular parameter values are listed in Table 1. If the membrane potential, V_m_, exceeds a voltage threshold, V_th_, then the neuron generates an action potential and releases neurotransmitter into the synaptic cleft. After generating an action potential, the pyramidal cell or interneuron enters an absolute refractory period, τ_ref_, which ends with the neuron returning to its resting potential, V_rest_. The leaky current was voltage-dependent is given by

$$I_{Leak}=g_{L}(V_{m}-V_{L})$$

where g_L_ is the membrane conductance and V_L_ is the leak reversal potential.

The current from NMDA receptors were modeled with the equation [35]

$$I_{NMDA}=\frac{g_{NMDA}s(V_{m}-V_{E})}{1+[Mg]e^{-0.062V_{m}}/3.57}$$

where g_NMDA_ is the receptor conductance when in the open state, s is the fraction of receptors in the open state, V_E_ is the synaptic reversal potential, and [Mg] is the concentration of Mg^2+^ ions in the extracellular fluid. The proportion of receptors in the open state, s, was controlled by a second-order kinetic, which is described by the equations

$$\frac{dx}{dt}=\alpha_{x}\sum_{i}\delta (t-t_{i})-x/\tau_{x}$$

$$\frac{ds}{dt}=\alpha_{s}x(1-s)-s/\tau_{s}$$

where i is the number of spikes, x is an intermediate gating variable, τ_x_ and τ_s_ are the mean lifetimes of the receptors changing from the closed-to-open and open-to-closed states, respectively, α_x_ is how much the x kinetic’s value changes with each received spike, and α_s_ controls the saturation of the receptor [35,36].

The currents from GABA receptors were simulated as having first-order kinetics. The current from GABA receptors was given by

$$I_{GABA}=g_{GABA}s(V_{m}-V_{I})$$

with s being the synaptic gating variable, g_NMDA_ being the receptor conductance, and V_I_ being the synaptic reversal potential. The parameter s was modeled as a first-order kinetic that increases by the weight of the connection with each presynaptic action potential and exponentially decreasing over time [23].

The afferent signal, I_Aff_, and current caused by noisy cell firing, I_Noise_, were modelled as AMPA-mediated Poisson processes [23]. The currents from AMPA receptors were controlled by the equation

$$I_{AMPA}=g_{AMPA}s(V_{m}-V_{E})$$

with s being the synaptic gating variable, g_AMPA_ being the receptor conductance, and V_E_ the synaptic reversal potential. The s kinetic was modeled as a first-order kinetic that increased by the weight of the connection from each spiking presynaptic pyramidal cell and exponentially decreasing. Excitatory postsynaptic potentials (EPSPs) caused by noisy firing were assumed to be uncorrelated with each other and occurred at a rate of 1.8 kHz. The afferent currents were also modeled as an uncorrelated Poisson process, but the strength of these currents were unevenly distributed. Afferent signals were localized by setting the afferent firing rate based on a neuron’s radial direction. The afferent firing rates were set according to

$$F(\Theta_{i}-\Theta_{Aff})=F_{Max}e^{2\pi \left(cos(\Theta_{i}-\Theta_{Aff})-1\right)/\sigma }$$

with Θ_i_ being the neuron’s radial direction, Θ_Aff_ being the radial direction associated with the stimulus, F_Max_ being the maximum firing rate, and σ as the width of the Gaussian-like distribution. The weight of each afferent and noise connection was 0.001. For all simulations, σ was set to 0.4 and F_Max_ was set to 1.25 kHz. AMPA receptor conductances from intracircuit connections were not modeled, because previous studies had found that activity-bumps could be maintained without them [23] and because they would entail unnecessary complications to the modeling of EI balance.

**Table 1:** Neuron Model Parameters.

Parameter	Value	Unit
**All Cells**		
V_th_	-50	mV
V_rest_	-60	mV
τ_ref_	2	mS
V_L_	-70	mV
*NMDA Receptors*		
V_E_	0	mV
[Mg]	1	mM
α_X_	1	ms^-1^
τ_X_	2	ms
α_S_	1	dimensionless
τ_S_	80	ms
*GABA Receptors*		
V_I_	-70	mV
τ_S_	10	ms
*AMPA Receptors*		
V_E_	0	mV
τ_S_	2	ms
Noise Firing Rate	1.80	kHz
**Pyramidal Cells**		
C_m_	0.50	nF
g_L_	25	nS
g_NMDA_	*	μS
g_GABA_	1.25	μS
Noise g_AMPA_	3.10	μS
Signal F_Max_	1.25	kHz
Signal g_AMPAμS_	*	μS
**Interneurons**		
C_m_	0.20	nF
g_L_	20	nS
g_NMDA_	*	μS
g_GABA_	1	μS
Noise g_AMPA_	2.38	μS

* = Experimentally varied

Changes in receptor kinetics and membrane voltages were integrated using the second-order Runge-Kutta method. Integrations were computed over intervals (dt) of 0.05 ms. Firing times were linearly interpolated [37]. All simulations and analyses were written in Python (v. 3.7.6) using the NumPy (v. 1.18.1), SKLearn (v. 0.22.1), MatPlotLib (v. 3.1.3), and SciPy (v. 1.4.1) modules.

### Ring attractors

Pyramidal cell and interneuron populations were paired and interconnected to create representational modules. Pyramidal cells were assigned radial directions that were evenly distributed between 0 and 2π radians. The weights from one neural population to another (or itself) were normalized such that the sum of connection weights to any given neuron from a population was 1. Connections from pyramidal cells to interneurons, interneurons to pyramidal cells, and interneurons to interneurons were non-localized (i.e., global) and had identical weights to all target neurons (solid line in Fig 2C). The excitatory connections between pyramidal cells were localized such that connections were stronger when two cells had a similar direction (dashed line in Fig 2C). The weights of pyramidal cell excitatory connections are given by

$$W(\Theta_{i}-\Theta_{j})=Pe^{2\pi \left(cos(\Theta_{i}-\Theta_{j})-1\right)/\sigma }+(1-P)$$

where Θ is the radial direction of a pyramidal cell, i is the presynaptic neuron, j is the postsynaptic neuron, P is the proportion of the connection weight that is controlled by a Gaussian-like distribution, and σ controls the width of the Gaussian-like distribution. The parameter values for all localized connections were P = 0.7 and σ = 0.05, which produces highly localized activity that is resistant to activity bump drift. This localization effectively discretizes the ring attractor into regions that can be associated with cues and probes. The authors do not believe that a ring attractor is necessary for this modeling, but we utilized it due to its benefits in finding network parameters.

### Representational modes of ring attractor firing

Ring attractors maintain representational information via activity bumps [38]. Each network was assumed to be in one of multiple states, which correspond with what information is represented within that network. During the cue-probe experiment, the network could be in one of three states, which were cue, probe, and no representation. During the DPX experiments, the memory and perception networks could be in one of five states, which were A-cue, B-cue, X-probe, Y-probe, and no representation. Which information is represented within a network can be determined by examining how similar the network’s current spiking activity is to the expected activity when it is representing specific-stimulus information [38,39]. The expected activity can be determined by creating tuning curves of when information is represented and aligning these to the vectors associated with each stimuli (e.g., π/2 for the cue and 3π/2 for the probe).

Tuning curves for each ring attractor were calculated from the network’s spiking activity during the last 100 ms of the cue presentation of all trials, which is when representation of the stimuli is most likely to exist in all networks given the constant external stimulation. A sliding 25-ms time window was taken over this period to determine the vector of the network’s spiking over that window, and the center of population activity was used to align network activity to the same direction. The vector of the population’s spiking activity was calculated by taking the arctangent of the mean y- and x-position, which were determined by taking the sine and cosine, respectively, of each spiking neuron’s associated direction and multiplying it by their firing rate during the interval. Since all of the simulated neurons are identical, the tuning curve was thus defined as

$$T=\frac{{\bar{F}}_{k}(\varphi )}{max{\bar{F}}_{k}(\varphi )}$$

where F_k_ is the firing rate in the population vector aligned direction φ. The resulting tuning curve was then smoothed with an 11-neuron-width boxcar filter.

How consistent the current spiking activity is with the expected activity can be determined by comparing the current activity bump with an expected activity bump [38]. The current activity bump was calculated at each ms via

$$A(x,t)=\frac{\sum_{k}\left[T_{k}(x)F_{k}(t)\right]}{\sum_{k}T_{k}(x)}$$

in which *k* is a neuron, F_k_ is the firing rate of neuron *k* divided by the maximum firing rate, and *x* is the radial orientation of neuron *k*. The time, *t*, was a 25-ms time window. The activity bump thus becomes normalized between 0 and 1, and is a smoothed representation of the spiking activity that can be compared to the expected activity. The expected activity for when a stimulus is being represented is similarly described by

$$\hat{A}(x)=\frac{\sum_{k}\left[T_{k}(x)T_{k}(\hat{x})\right]}{\sum_{k}T_{k}(x)}.$$

A measure of the distance from the actual and expected activity bump can be calculated by taking their sum of squares differences.

A representational similarity index was used to determine which stimulus was best represented in the network and how different it was from other potential representations. The distance of the current activity bump from the expected activity bumps of each stimulus were calculated. Dividing the distance from each stimulus distance by the mean of the other stimuli distances provides an index of which stimulus best represents the network. When the index is less than 1, then the distance of the current activity bump from that stimulus’ expected activity bump is better than other stimuli (i.e., the best description). We used an index cutoff of 0.75 for when information was represented to permit for noise around an index of 1.0, which would indicate no difference between that stimuli and other stimuli. The initiation time of representation was defined as when the the network was best and continuously described by a single stimuli’s expected activity bump representation for 50 ms.

### Cue-Probe experiment

Cue-probe stimulus pairings were used in a series of experiments to assess changes in activity-bump representation during the first two experiments (Fig 2A). Each network experienced 100 cue-probe trials, and the network was simply reset at the end of each trial because previous experiments have shown that it is relatively easy to eliminate persistent neural activity [22,23]. After an initial 500 ms delay, a cue was presented for 500 ms. A 2000 ms interstimulus interval was followed by 500 ms of probe presentation and a 500 ms intertrial interval. The cue stimulus was associated with the radial direction of π/2 and the probe with 3π/2. During stimuli presentations, the afferent current to the pyramidal cells was turned on. Θ_Aff_ of the cue stimulus was π/2 and the probe stimulus was 3π/2.

### Dot pattern expectancy (DPX) task

The DPX task assesses context integration by presenting participants with a series of cue-probe pairings (with an interval between them) that require different actions depending upon the pairing. The DPX task [13] was simplified to its core components for this simulation. Each trial consisted of a cue presentation, followed by a delay, and a probe presentation (Fig 1). Typically, the cues and probes can be one of any six stimuli but the participant is meant to identify a specific pair of these, which are typically referred to as the A cue and the X probe. If both the A cue and X probe are presented then the participant is instructed to perform the ‘target’ response, which is a joystick pull to the left. For all other cue and probe combinations, the participant is instructed to perform the ‘non-target’ response, which is a joystick pull to the right. For the sake of simplicity, the 5 distractor cues were consolidated into a single B cue and the 5 probe distractors were consolidated into a single Y probe. The radial directions associated with each stimulus were: A = 0.3π, B = 0.7π, X = 1.3π, and Y = 1.7π. The differences with this task and the simple cue-probe experiments was that there are two cue stimuli, two probe stimuli, the interstimulus interval was longer, and correct actions depended upon the specific cue-probe pair.

The trial structure was minimally altered for our experiments (Fig 5B shows an example of an AY trial). The typical duration of the ITI is 1100 ms, which we split between the beginning and end of each trial. For the first 500 ms of each trial the network did not receive any afferent stimulation. At 500 ms, the network received afferent stimulation that corresponded with either the A or B cue for 1000 ms. A 4000-ms ISI followed the cue before the probe presentation. A brief 250-ms distractor was presented in the middle of the ISI to ensure that the perception network was not maintaining the memory network. Without the addition of a distractor some effects of the NMDA conductance manipulation were difficult to observe (compare Figs 5D, 5E, and 5F to S1). The distractor was randomly presented at either 0 or π, which were not associated with the experimental stimuli. At 5500 ms into the trial, the network receives afferent stimulation that corresponds with either the X or Y cue for 500 ms. Once the probe was presented the agent made either a ‘left’ or ‘right’ action, and the probe was followed by the first half of the ITI (600 ms). The network was simply reset at the end of each trial, because no learning capacity was built into the model and previous simulations have indicated methods for resetting activity bumps [22,23].

### The DPX agent

The DPX agent coordinated perception and memory of stimuli, via ring attractors, to determine its actions. The parameters for the perception and memory rings were selected based upon their inertia to activity bump jumping (P & M in Fig 3). The NMDA receptor conductance parameter values for the pyramidal cells and interneurons of the perception network were 0.37 μS and 0.30 μS, respectively. Similarly, the memory network’s NMDA receptor conductance parameter values were 0.37 μS and 0.35 μS for the pyramidal cells and interneurons, respectively. This set of parameter values for the DPX agent will henceforth be referred to as the neurotypical agent. The transition from maintaining the current activity bump to switching to the probe activity-bump representation, inertia, were at afferent conductances of 0.8 μS for the perception network and 1.1 μS for the memory network.

Stimulus information was fed forward through the agent’s architecture (Fig 4A). Stimulus information entered the agent via an afferent current to the pyramidal neurons of the perception ring attractor. The AMPA-mediated receptor conductance of this afferent current was set to 1.0 μS, which was above the perception network’s inertia to change. Firing of pyramidal cells in the perception network fed stimulus information forward to the pyramidal cells of the memory network. These weights were localized, but relatively weak; the sum of connection weights to any given pyramidal cell of the memory network from the perception network was 0.05. This was substantially less than the normalization to 1 for the ring-attractor intraconnections, and was weak enough to ensure that the neurotypical agent’s memory network would not easily jump from the cue representation to the probe representation.

The information that was represented within the perception and memory networks was used to determine the agent’s actions. If the similarity index of the network’s current activity to a stimulus was less than 0.75, then it’s degree of similarity was constantly fed forward to the two response accumulators. We used the representational similarity index as a proxy for the relative strength of the current stimulus against other possible stimuli representations. The current similarity index value was subtracted from 1, then multiplied by a weight parameter, and finally added to one of two response accumulators. The response accumulators were used to determine the likelihood of the agent making a left or right action. These response accumulators were assumed to be activated through an NMDA-mediated process, which was simulated as a simple first-order kinetic that increased by the connection weight from the perception or memory network when a EPSP was produced and exponentially decayed with a mean lifetime of 80 ms. The connections from perception and memory (Fig 4A) were weighted to favor the ‘right’ (i.e., not-target) response with weights of 1.25 from the memory circuit while it represented the B cue (activity bump at 0.7π) and 2.00 from the perception circuit while it represented the Y probe (activity bump at 1.7π). The connections to the ‘left’ (i.e., target) response accumulator had weaker weights of 1.00 from the memory circuit while it represented the A cue (activity bump at 0.3π) and 0.25 from the perception circuit while it represented the X cue (activity bump at 1.3π). These weights were smaller than from the non-target response because B and Y stimuli require that the agent override the action suggested by A and X stimuli. These weights were chosen to reflect the pattern of deficits observed in humans when they do not perform well on the DPX task (see S2 Fig for the rationale and supporting information).

To translate the response accumulation into action, softmax [40] was combined with a drift-diffusion-esque model (DDM) that was used to create the probabilities of engaging in a left or right response from two response accumulators. At every time step, softmax set the agent’s action probabilities via

$$P(a)=\frac{e^{s_{a}/\tau }}{\sum_{i}e^{s_{i}/\tau }}$$

where a is the action, s is the first-order kinetic’s value, i is all possible actions, and τ controls how strongly the larger kinetic determines the action. The parameter τ was set to 15, which created a strong preference for the response accumulator with the greater value. This softmax step was necessary, because there is no linear combination of weights that generates correct responding. This step can be thought of as creating two attractor states for each potential action.

The response accumulation and softmax steps created a number of features that would be expected in a DDM (Fig 4B) [28,29]. When reaching the probe period the response process was initiated when the perception network represented either an X or Y probe, which is different from the probe’s starting time, and is analogous to the start-time (*t*) parameter when fitting a DDM. The agent’s response could be biased (*z* parameter in a DDM) towards either a left or right action due to the influence of the cue on decision making. The representational information from the perception network altered the agent’s likelihood of engaging in a left response over time, which is similar to the drift-rate parameter (*v*) in a DDM. We utilized noisy left and right collapsing decision-boundaries due to the network probability of response being between 0 and 1, which were analogous to the *a* parameter in DDM models but included the noise that is typically added to the accumulation process. The intercept and slope of the collapsing boundaries were independently randomized, which resulted in faster collapsing thresholds for either the left or right response on any given trial. Once either decision-boundary crossed the response probability, which was evaluated at every ms, the agent engaged in the crossed decision-boundary’s associated action at that time. The left decision boundary’s starting threshold was selected from a Gaussian distribution with a mean of 1.15 and standard deviation of 0.075. The default slope was set to reach a response probability of 0 at 500 ms after probe representation, but was multiplied by a random Gaussian with a mean of 1.0 and a standard deviation of 0.45. Since the probability of a right response was one minus the probability of a left action, the right decision boundary was a mirror of this process and started at threshold less then 0 (Fig 4B). If a response was not chosen by the end of the trial, the thresholds and slopes were resampled. The overall decision making process is most akin to a leaky integration model [41,42] as the accumulators are primarily influenced by recent information and compete with another to determine the action, which has been argued to be similar to how the attractor dynamics of a LIF based agent stochastically selects responses [41].

## Supporting information

S1 Fig Task Error Rates Without ISI Distractors. (TIF)

S2 Fig Across Study Metaanalysis of the Relationships Between the Error Rates on Different Trial Types. (TIF)
