# Supplementary figures and images for: Global disruption in excitation-inhibition balance can cause localized network dysfunction and Schizophrenia-like context-integration deficits
Source: PLoS Comput Biol. 2021 May 25;17(5):e1008985. doi: 10.1371/journal.pcbi.1008985 (PMC8184155; doi:10.1371/journal.pcbi.1008985)

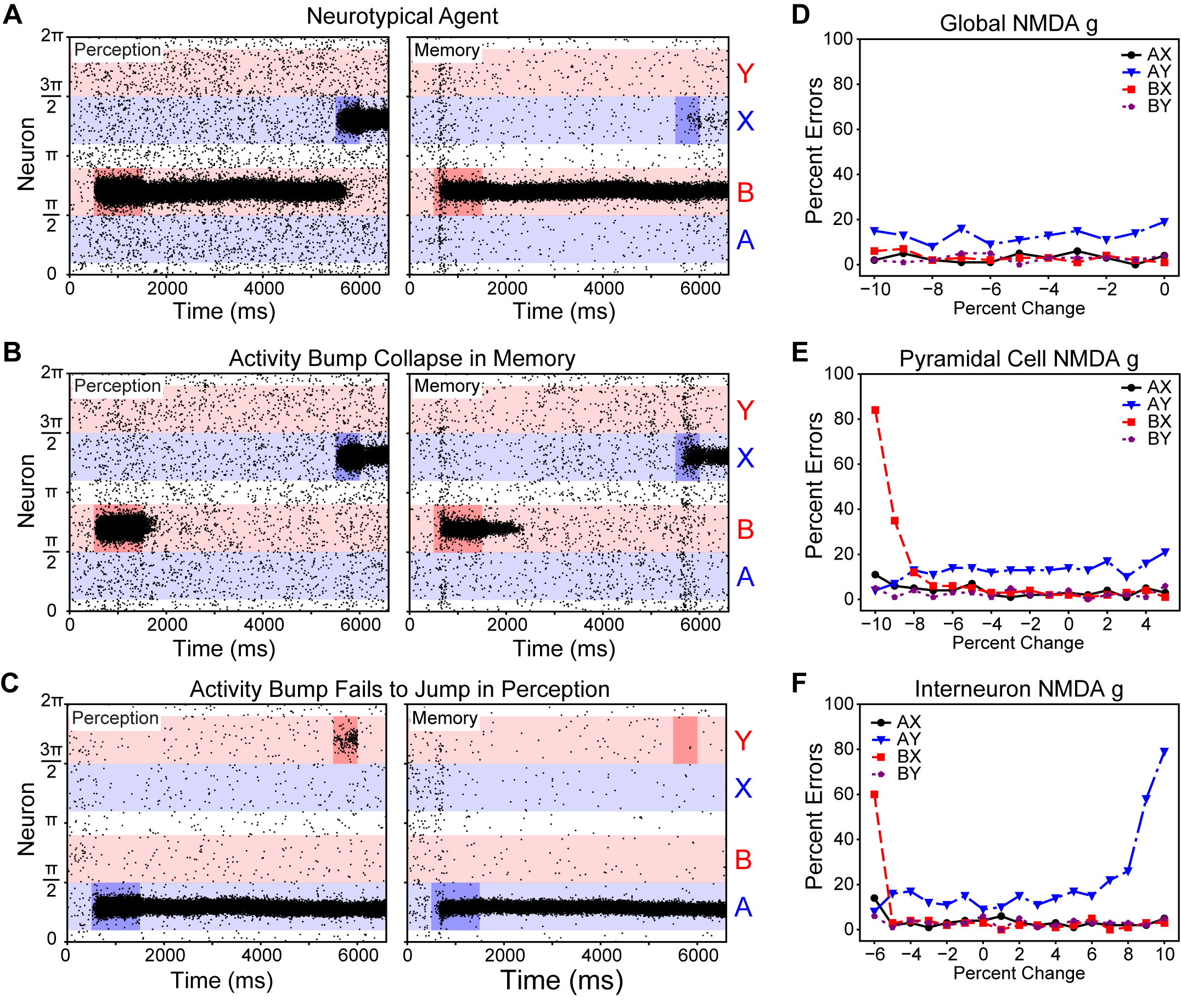

Supplement: S1 Fig — (TIF) [file pcbi.1008985.s001.tif]

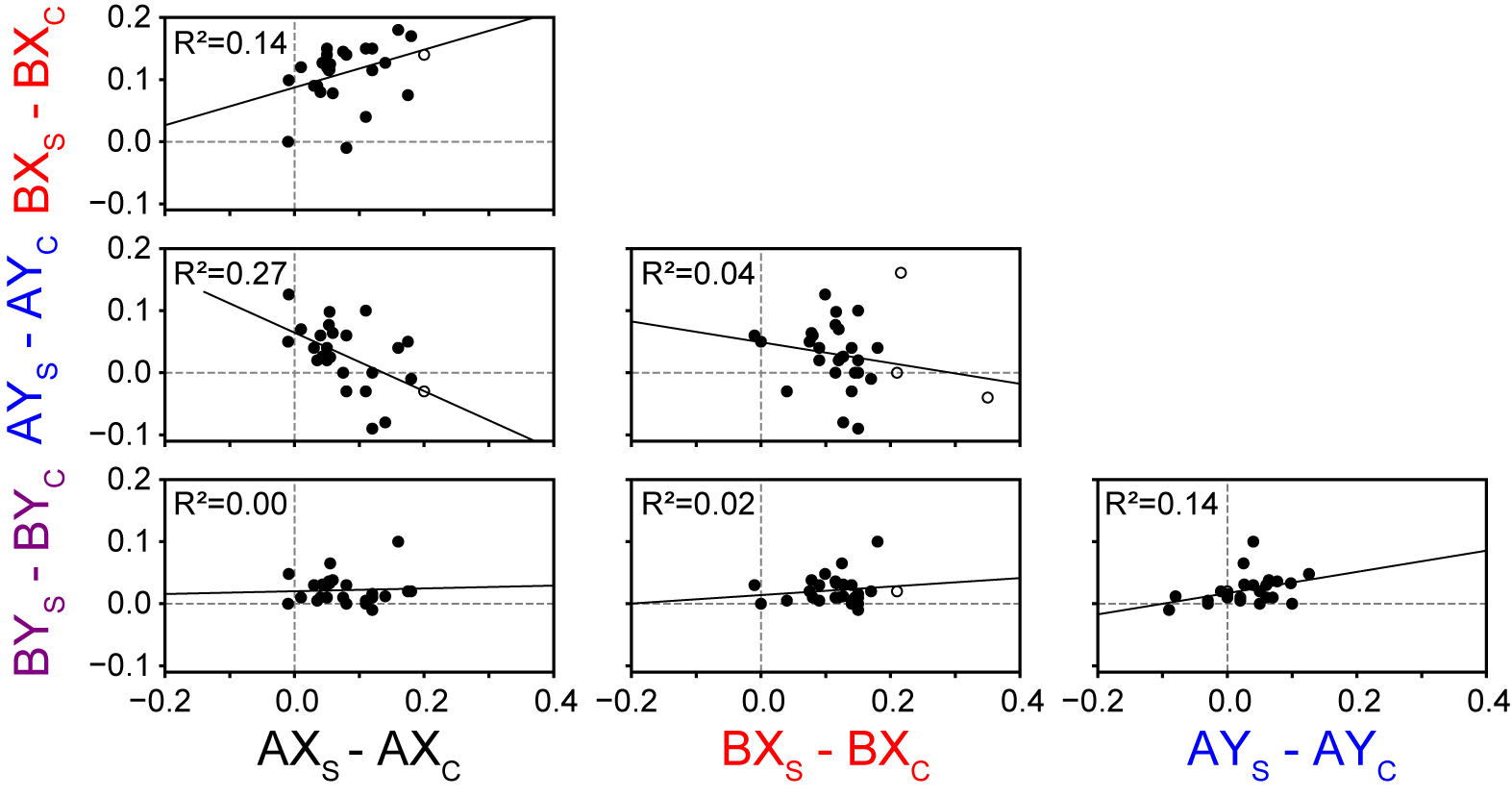

Supplement: S2 Fig — (TIF) [file pcbi.1008985.s002.tif]
